# Frailty or frailties: exploring frailty index subdimensions in the English Longitudinal Study of Ageing

**DOI:** 10.1136/jech-2023-221829

**Published:** 2024-07-23

**Authors:** Lara Johnson, Bruce Guthrie, Paul A T Kelly, Atul Anand, Alan Marshall, Sohan Seth

**Affiliations:** 1 The University of Edinburgh School of Engineering, Edinburgh, UK; 2 Advanced Care Research Centre, University of Edinburgh, Edinburgh, UK; 3 The University of Edinburgh Usher Institute of Population Health Sciences and Informatics, Edinburgh, UK; 4 The University of Edinburgh School of Social and Political Science, Edinburgh, UK; 5 The University of Edinburgh School of Informatics, Edinburgh, UK

**Keywords:** GERIATRICS, HEALTH, MORBIDITY, QUALITY OF LIFE, AGING

## Abstract

**Background:**

Frailty, a state of increased vulnerability to adverse health outcomes, has garnered significant attention in research and clinical practice. Existing constructs aggregate clinical features or health deficits into a single score. While simple and interpretable, this approach may overlook the complexity of frailty and not capture the full range of variation between individuals.

**Methods:**

Exploratory factor analysis was used to infer latent dimensions of a frailty index constructed using survey data from the English Longitudinal Study of Ageing, wave 9. The dataset included 58 self-reported health deficits in a representative sample of community-dwelling adults aged 65+ (N=4971). Deficits encompassed chronic disease, general health status, mobility, independence with activities of daily living, psychological well-being, memory and cognition. Multiple linear regression examined associations with CASP-19 quality of life scores.

**Results:**

Factor analysis revealed four frailty subdimensions. Based on the component deficits with the highest loading values, these factors were labelled ‘mobility impairment and physical morbidity’, ‘difficulties in daily activities’, ‘mental health’ and ‘disorientation in time’. The four subdimensions were a better predictor of quality of life than frailty index scores.

**Conclusions:**

Distinct subdimensions of frailty can be identified from standard index scores. A decomposed approach to understanding frailty has a potential to provide a more nuanced understanding of an individual’s state of health across multiple deficits.

**WHAT IS ALREADY KNOWN ON THIS TOPIC**

- A single frailty score may overlook the complexity of frailty and not capture the full range of variation between individuals. Despite being recognised as an early research priority in the field, only a limited number of studies have explored the dimensionality of frailty, and various subdimensions of a range of frailty measures have been proposed for different subgroups of the population.

**WHAT THIS STUDY ADDS**

- Factor analysis revealed four frailty subdimensions: (1) mobility impairment and physical morbidity, (2) difficulty in daily activities, (3) mental health and (4) disorientation in time. Only ‘mental health’ is closely aligned with previously documented factors in the literature. ‘Mobility impairment and physical morbidity’ and ‘difficulties in daily activities’ shared some similarities with factors from previous studies but also exhibited significant differences. ‘Disorientation in time’ was not encountered in previously studies, even those incorporating items on memory and cognition. These four subdimensions were a better predictor of CASP-19 quality of life scores than frailty index scores. Crucially, our analysis is the first analysis on a frailty index based on a large (N=4971) representative sample of both men and women of all ages over 65.

**HOW THIS STUDY MIGHT AFFECT RESEARCH, PRACTICE OR POLICY**

- Frailty subdimensions can provide a more nuanced understanding of an individual’s state of health across multiple deficits. Future research is needed to develop clinical guidelines and establish thresholds for frailty, with detailed comparison of factor scores against outcomes such as hospitalisations, falls, fractures and mortality.

## Introduction

Rapidly ageing populations challenge healthcare systems internationally because of the increasing number of people living with multimorbidity and frailty.^1^ Frailty describes a state of increased vulnerability to adverse health outcomes for individuals compared with their peers of the same age.^2^ Frailty is associated with accelerated functional decline, higher mortality, increased hospital admissions, long-term care stays, primary and secondary care costs and lower quality of life.^3–5^

There are several approaches to frailty assessment, including the phenotype model (defined by the presence of clinical features),^6^ the Rockwood Clinical Frailty Scale (based on functional ability)^7^ and the cumulative deficit model, which counts health deficits to calculate a frailty index as the proportion of possible deficits present in an individual.^8^ The frailty index is a widely used instrument for identifying frail older adults from secondary data and can be constructed from both electronic health records and survey data.^9^ A higher frailty index score is associated with an increased risk of experiencing an adverse health event.^10^ A distinctive feature of the cumulative deficit model of frailty is that frailty index scores can be automatically calculated using existing data.^10^ This makes it possible to evaluate (or at least screen for) the presence of frailty across entire populations.^3^

These existing constructs all represent frailty with a single score or binary status. The advantage of this approach is its simplicity. Frailty scores are a straightforward and easy-to-understand measure of frailty. The frailty index, in particular, is easy to compute (adding up counts) and does not require weights to be calibrated across different populations.

However, frailty is a complex syndrome that encompasses a range of physical, cognitive and psychosocial factors.^11^ There can be considerable variation between individuals with the same frailty index score. These variations are especially apparent between different demographic groups. Women are more frail than men^12^ but live longer.^13^ Women have a lower risk of mortality, even at the same frailty index scores,^12^ but have a higher risk of falls^14^ and hip fractures.^15^ This suggests that a single score may overlook the complexity and possible multidimensionality of frailty and not capture the full range of variation between individuals.

The identification of frailty subdimensions was recognised as a research priority in 2006.^16^ Since then, however, few studies have explored subdimensions of frailty. One,^17^ two^18^ and three^19^ dimensions of the phenotype model of frailty have variously been suggested. Seven factors of frailty indicators^20^ and ordinal multimorbidity items^21^ have been proposed.

This study aimed to explore empirically observed patterns in health deficits to identify distinct subdimensions of frailty and assess them for their ability to explain quality of life. Quality of life was selected as the outcome measure due to its alignment with the concerns and priorities of older individuals, as underscored by our public contributor, and its established relevance in prior research on frailty in community-dwelling older people.^5^

## Methods

### Data source

The study population was drawn from the 7289 participants in wave 9 of the English Longitudinal Study of Ageing (ELSA), a population-based study of community-dwelling adults aged 50 years and over living in England.^22^ We excluded participants aged <65 years (N=2314) and with missing data for more than 20 deficits (N=4), resulting in a final sample of 4971.

### Measures

We used the frailty index developed in a previous study^23^ for use in ELSA, which comprises 58 deficits across a range of chronic diseases (eg, hypertension), general health (eg, vision impairment), mobility (eg, getting up from a chair), activities of daily living (eg, ability to get dressed), psychological well-being (eg, sadness) and memory (online supplemental materials table 1). We inspected each of these deficits against the standard criteria for creating a frailty index^9^ to explore population prevalence, saturation by age group and correlation with increasing age.

10.1136/jech-2023-221829.supp1 Supplementary data

We used Control, Autonomy, Self-Realisation and Pleasure (CASP-19) to assess quality of life.^24^ CASP-19 is a self-report measure developed for older adults and comprises 19 items spanning CASP-19,^25^ with composite scores ranging from 0 to 57 and higher scores representing better quality of life.

### Statistical analysis

Our fundamental assumption is that frailty is a latent state or a set of latent states that manifests in the observable deficits. Consequently, we adopt a latent variable model to describe frailty wherein the latent factors act as drivers for the observed variables. We performed exploratory factor analysis to determine how many factors are necessary to adequately explain the patterns of correlations among observed deficits and to estimate the loadings of each variable on the latent factors. We used the factor loadings to interpret the meaning of each factor based on the variables with high loadings and to consider their implications from a clinical perspective.

We opted for the phi coefficient^26^ as our measure of association between two binary variables since some tetrachoric correlations could not be estimated due to the contingency table having zero diagonal cells.^27^ Parallel analysis and the visual scree test were used to determine the appropriate number of factors to retain.^28^ Parsimony and theoretical meaningfulness were also considered. Acceptable model fit is indicated by a root mean square of the residuals (RMSR) of 0.05 or less. We employed an oblimin rotation, as it was assumed that the factors would be correlated. The criteria for determining factor adequacy were established a priori. Factor loadings >0.20 or <−0.20 were considered salient. Factors with a minimum of three salient pattern coefficients and that were theoretically meaningful were considered adequate. We conducted Pearson’s correlation analysis to examine the relationships between the factor scores as well as with the frailty index score.

Two multiple linear regression models were fitted to examine their effectiveness in explaining the association between frailty and quality of life. Both models included age and sex (0=male, 1=female) as independent variables and the CASP-19 score as the dependent variable. Additional independent variables were the frailty index score in model 1 and the four factor scores in model 2. All variables were standardised. We compared the two models’ R^2^ scores.

Analyses were performed by using R (V.4.3.1)^29^ and its psych package (V.2.3.3).^30^ Missing data were handled using pairwise deletion.^31^

## Results

The study population comprised 4971 people aged 65 years and older (table 1), of which 56.6% were female. The mean age was 74.9 years (SD=7.0). 95.4% of respondents had at least one deficit. The mean number of deficits was 7.68 (SD=6.96) and the mean frailty score was 0.13 (SD=0.12). The mean CASP-19 score was 42.64 (SD=8.11).

**Table 1:** Study population characteristics

	Men		Women		Total	
	N	%	N	%	N	%
N	2156	43.4%	2815	56.6%	4971	
Age (years)						
65–69	563	26.1%	731	26.0%	1294	26.0%
70–79	1052	48.8%	1293	45.9%	2345	47.2%
80–89	483	22.4%	670	23.8%	1153	23.2%
90+	58	2.7%	121	4.3%	179	3.6%
Number of deficits						
0	122	5.7%	108	3.8%	230	4.6%
1–5	1145	53.1%	1178	41.8%	2323	46.7%
6–10	464	21.5%	679	24.1%	1143	23.0%
11–15	200	9.3%	376	13.4%	576	11.6%
16+	225	10.4%	474	16.8%	699	14.1%
Number of deficits, mean (SD)	6.56 (6.29)		8.54 (7.31)		7.68 (6.96)	
Frailty Index Score, mean (SD)	0.113 (0.109)		0.147 (0.126)		0.132 (0.119)	
CASP-19 score, mean (SD)	42.80 (7.90)		42.50 (8.27)		42.64 (8.11)	

CASP-19, Control, Autonomy, Self-Realisation and Pleasure.

The most common deficit experienced by all participants was arthritis (48.8%) and the least was having a diagnosis of Alzheimer’s disease (1.0%) (online supplemental material table 2). None of the deficits were universally prevalent at any age. The deficit with the highest prevalence in a single age group was difficulty climbing several flights of stairs without resting, which was present in 80.3% of respondents aged over 90. Of the 58 deficits, 53 were moderately or strongly correlated with age (r>0.25). The mean correlation with age was r=0.66. The deficit most correlated with age was climbing several flights of stairs (r=0.92). The deficits with weak or negative correlations with age were lung disease (r=0.04), asthma (r=−0.27), restless sleep (r=−0.38), pain while walking (r=−0.44) and having any psychiatric condition (r=−0.67).

Parallel analysis and visual inspection of the scree plot suggested that four factors should be retained. Through factor analysis with the oblimin rotation method, we extracted four factors. The four factors had eigenvalues of 10.9 (12%), 3.4 (9 %), 2.4 (5%) and 1.9 (2%), respectively. The RMSR was 0.02, indicating an acceptable model fit.

**Figure 1:**
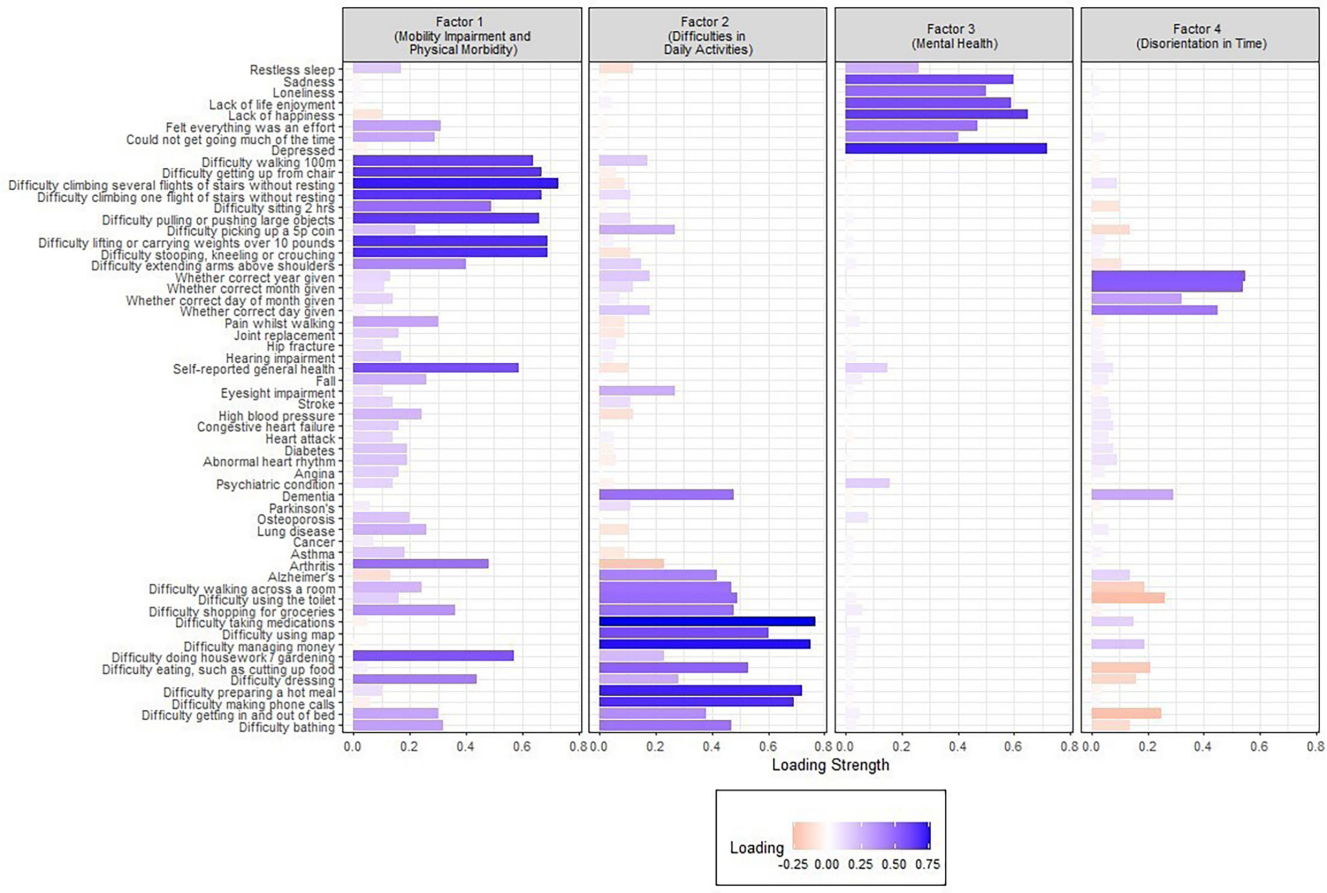
Factor loadings.

The factor loadings of the deficits, which represent the strength and direction of the relationship between observed variables and the latent factors, are shown in figure 1.

Factor 1 was saliently loaded by 25 items, including difficulty climbing several flights of stairs without resting (0.73), kneeling (0.69), lifting 10 pounds (0.69), getting up from a chair (0.67), walking for 100 m (0.64) and performing various other movements. It also emphasised the burden of chronic diseases such as arthritis (0.48), hypertension (0.24) and osteoporosis (0.20). We labelled this factor ‘mobility impairment and physical morbidities’, given that the largest loadings were for a range of physical mobility limitations and because health-related issues exhibited stronger loadings on this factor compared with any other factor.

Factor 2 was saliently loaded by 18 items, including difficulty taking medications (0.77), managing money (0.75), cooking (0.72), making phone calls (0.69), having a diagnosis of dementia (0.48) and poor eyesight (0.27). Items included basic self-care, such as difficulty using the toilet (0.49) and bathing (0.47) as well as instrumental activities of daily living (IADLs), such as difficulty shopping for groceries (0.48). IADLs are higher-level activities that individuals typically need to perform to live independently and that generally involve cognitive and organisational skills in addition to physical fitness. Arthritis, which had a strong positive loading on Factor 1 (0.48), had a low negative loading (−0.23) on this factor. Restless sleep (−0.12) negatively loaded onto this factor. We named this factor ‘difficulties in daily activities’.

Factor 3 was saliently loaded by eight items, including depression (0.72), sadness (0.60), feelings of loneliness (0.50) and sleep disturbances (0.26). We labelled this factor ‘mental health’.

Factor 4 was saliently loaded by eight items, including incorrectly recalling the year (0.55), the month (0.54) and the day of the month (0.45). Dementia loaded more highly onto Factor 2 (0.48) than this factor (0.28). Factor 4 had several negative loadings, including difficulty using the toilet (−0.26), getting in and out of bed (−0.25) and eating (−0.21). We named this factor ‘disorientation in time’.

**Figure 2:**
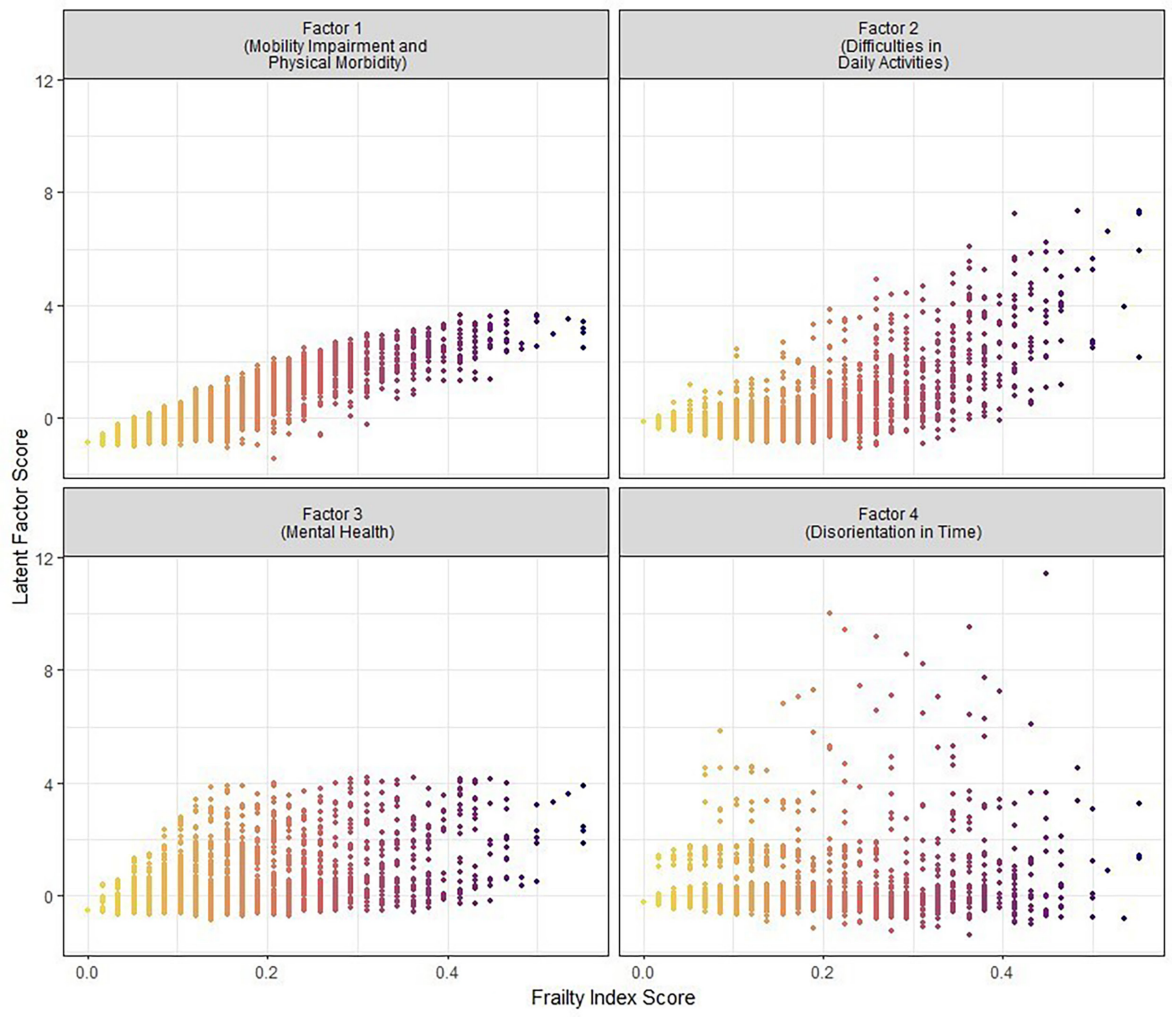
Comparison of individuals’ latent factor scores and frailty index scores, illustrating the variation in frailty subdimension for each frailty index score.

The factors were not mutually exclusive, and an individual can have a high score on more than one frailty subdimension. The Pearson correlations between factor scores ranged from 0.118 (Factor 3 and Factor 4) to 0.664 (Factor 1 and Factor 2). All factor scores correlated positively with the frailty index score (figure 2), ranging from r=0.294 for Factor 4 to r=0.929 for Factor 1.

**Figure 3:**
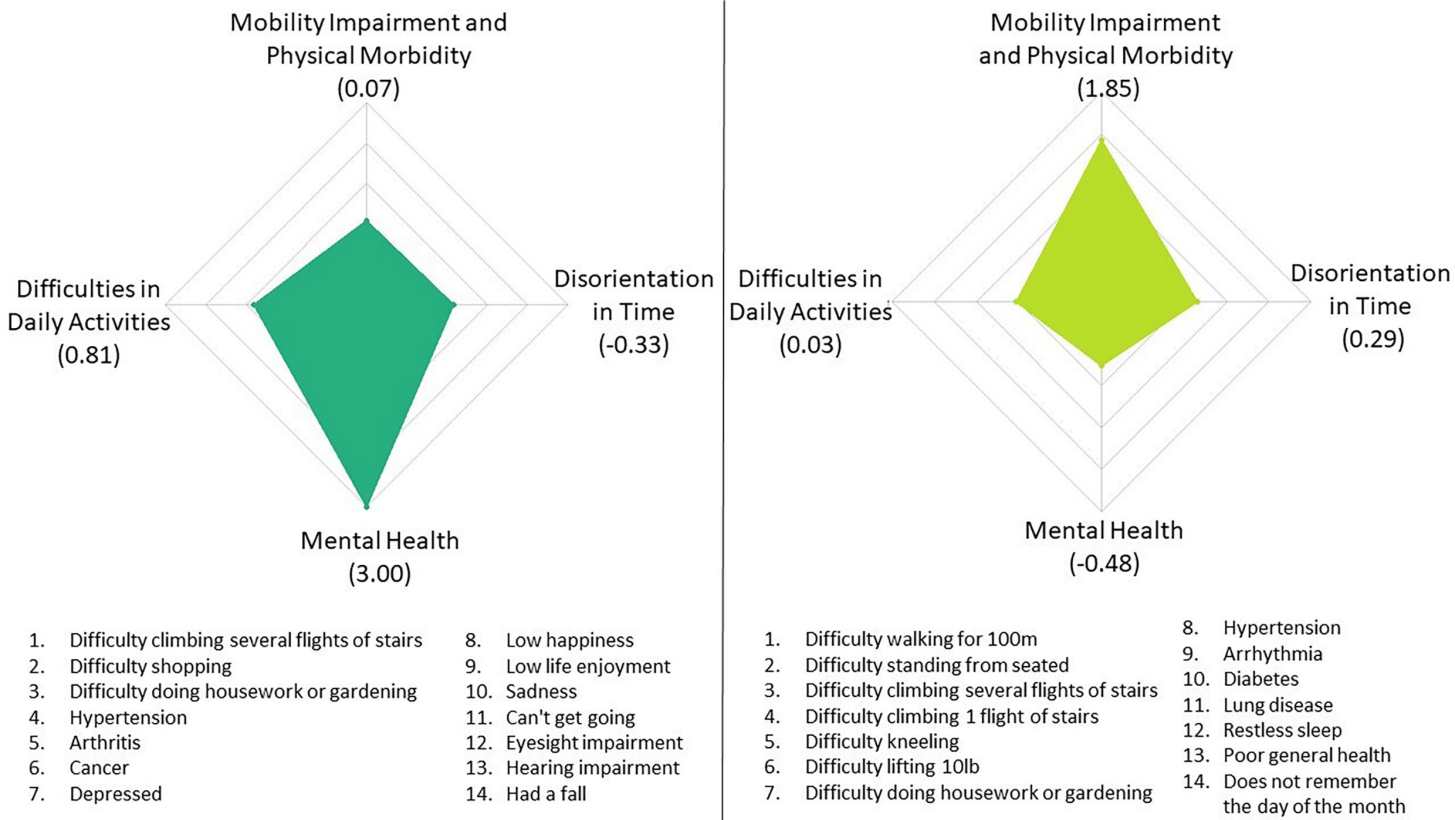
Illustration of frailty subdimensions in two individuals with the same frailty index score (0.24), matched for age group (80–85) and sex (F).

The diversity of frailty subdimensions can be illustrated by comparing two women in their early 80s with an identical frailty index score of 0.24. This single score denotes a moderate level of frailty but considering the frailty subdimensions reveals distinct profiles (figure 3): one displays an elevated ‘mental health’ score, whereas the other presents a heightened ‘mobility impairment and physical morbidities’ score.

**Figure 4:**
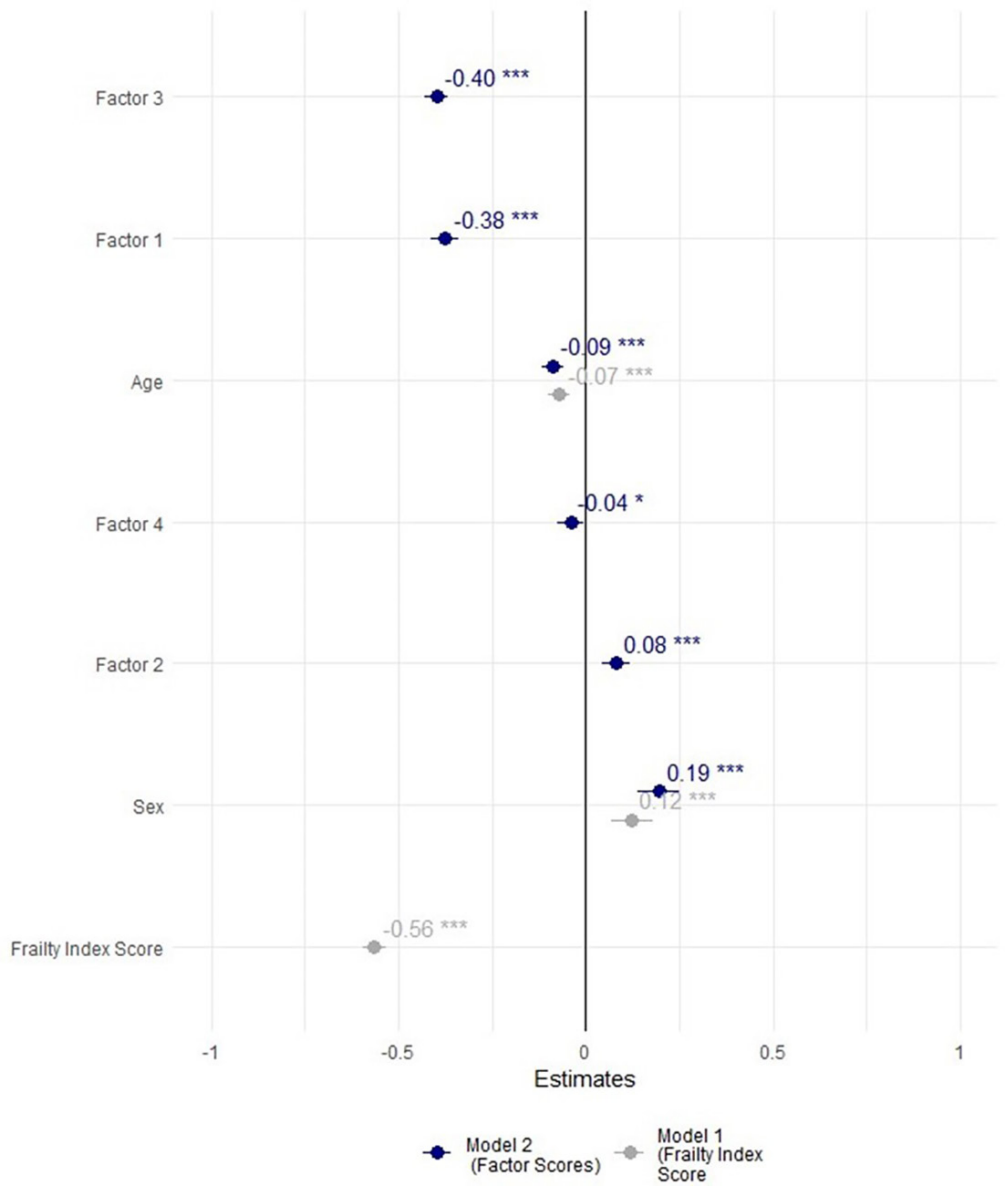
Forest plot of two multiple linear regression models examining the association between Frailty Index Scores (model 1) and factor scores (model 2) with quality of life.*p<0.05; **p<0.01; ***p<0.001.

The results of two multiple linear regression models (figure 4) revealed that factor scores (model 2) were a better predictor of quality of life than frailty index scores (model 1). Model 2 exhibited a higher explanatory power (R^2^=0.3538, adjusted R^2^=0.3527, p≤0.001) compared with model 1 (R^2^=0.3008, adjusted R^2^=0.3002, p≤0.001).

In model 2, sex (β=0.19, t=7.01, p<0.000) and Factor 2 (β=0.08, t=4.12, p<0.001) were positively associated with quality of life, signifying that being female and higher Factor 2 scores were linked to increased quality of life. Factor 3 (β=−0.40, t=−26.28 p<0.000), Factor 1 (β=−0.37, t=−19.70, p<0.001), age (β=−0.08, t=−6.16, p<0.001) and Factor 4 (β=−0.04, t=−2.35, p<0.01) exhibited negative associations. In substantive terms, this implies that a 1 SD increase in Factor 1 corresponds to a 0.37 SD reduction in quality of life.

## Discussion

Our factor analysis of the standard frailty index in ELSA identified four subdimensions: ‘mobility impairment and physical morbidities’, ‘difficulties in daily activities’, ‘mental health’ and ‘disorientation in time’. The correlation between these subdimensions was low, except for a moderate association between ‘mobility impairment and physical morbidities’ and ‘difficulties in daily activities’. Individuals’ overall frailty index core was largely driven by these factors, with ‘mental health’ and ‘disorientation in time’ showing a weaker association and suggesting that these subdimensions may not be sufficiently highlighted in a single frailty score. The four subdimensions outperformed frailty index scores in explaining associations with quality of life.

The strengths of this study are the large number of participants in a representative sample of the population and the availability in ELSA of a wide range of potential deficits including rich information on function, well-being and mental health, which are less robustly captured in routine health data datasets. ‘Mental health’ in particular appears differentiated from the other subdimensions observed in this study, with significant loadings only for psychological symptoms, depression, overall general health and the presence of psychiatric conditions. ‘Difficulties in daily activities’ capture a different set of difficulties than physical mobility impairments, which could be a useful distinction. There is precedent for using a frailty index on ELSA data to predict mortality,^32^ assess determinants of frailty^33^ and examine cohort differences in levels and trajectories of frailty.^23^

The study has several limitations. First, the reliance on a single data source may limit generalisation to other populations, such as care home residents not included in ELSA. Second, the cross-sectional nature of the study restricts the ability to discern temporal patterns or causal relationships. The factors recovered by the model are dependent on the measure of association used. Although phi-correlation is one choice of metric,^34^ another suitable alternative is tetrachoric correlation.^27^ While there is established precedent for investigating the relationship between CASP-19 and the frailty index,^35^ we acknowledge their item-level similarities. Finally, a multidimensional approach to frailty creates practical challenges for clinical application. Integrating and interpreting data from multiple dimensions requires careful consideration of their inter-relationships and potential interactions.

The existing literature offers different perspectives on the dimensionality of frailty (online supplemental material table 4). Two studies examined Fried’s phenotype model, proposing one^17^ and two^18^ subdimensions. However, the utility of factor analysis may be limited, given that the phenotype model consists of only five components. Another study^19^ evaluated the dimensionality of the 15-item Groningen Frailty Indicator (GFI) questionnaire, which incorporates various frailty indicators but does not include specific diseases. The study proposed three dimensions: daily activities, psychosocial functioning and health problems.^19^

Two studies explored the dimensionality of a frailty index.^20 21^ One study^20^ explored the dimensionality of 35 binary frailty indicators in a UK sample of 4286 women aged 60–79 years while the other used factor analysis on 30 ordinal multimorbidity items in a Canadian sample of 649 adults.^21^ Both studies identified seven distinct factors, though there were variations in the nature of these factors. Four of the factors consistently aligned across the studies: cardiac symptoms, respiratory symptoms, psychological problems^20^/emotional well-being^21^ and comorbidities. Physical ability and physiological measures^20^ approximately corresponded to physical activity and mobility.^21^ However, visual impairment^20^ and instrumental health^21^ were distinct.

Our study discovered four subdimensions that explained the relationship among 58 frailty index items. Only one dimension is closely aligned with previously documented factors in the literature. ‘Mental health’ corresponded to psychosocial functioning,^19^ psychological problems^20^ and emotional well-being.^21^ This convergence of findings across different studies underscores the consistency and relevance of the mental health dimension in frailty research.

Two subdimensions share some similarities with factors from previous studies but also exhibit significant differences. ‘Mobility impairment and physical morbidities’ amalgamates several dimensions identified in prior literature: mobility,^21^ physical activity,^21^ cardiac symptoms,^20 21^ respiratory symptoms,^20 21^ health problems^19^ and comorbidities,^20 21^ indicating a close interconnection between physical mobility and morbidities. ‘Difficulties in daily activities’ resemble the GFI ‘daily activities’ subscale^19^ but includes IADLs as well as basic self-care.

One of our subdimensions is entirely different. ‘Disorientation in time’ was not encountered in previous studies, even those that incorporated items on memory complaints^19^ or problems.^20^

While quality of life generally worsens with frailty,^5^ frailty subdimensions impact it differently. The positive association between female sex and improved quality of life underscores sex differences in frailty outcomes. ‘Mental health’ and ‘mobility impairment and physical morbidities’ contribute to reduced quality of life, outweighing the effect of increasing age. The small positive association of ‘difficulties in daily activities’ challenges conventional assumptions about the role of IADLs in overall well-being. The small negative association of ‘disorientation in time’ indicates that memory issues have a relatively minor influence on quality of life.

The presence of numerous items within a frailty index presents an ideal opportunity for consolidating into subdimensions. However, it is crucial to acknowledge that the specific items included in frailty indices can vary widely across different studies and populations (online supplemental material table 5). The absence of a single, uniform frailty index means that results may not be directly transferable from one study to another. This underscores the need for caution and rigour when comparing and generalising results across studies.

An important next step in advancing our understanding of frailty subdimensions is to replicate this work by examining frailty indices constructed from different data sources, including routine electronic health records. Future research is needed to develop clinical guidelines and establish thresholds for frailty, with detailed comparison of factor scores against outcomes such as hospitalisations, falls, fractures and mortality.

Taking into account subdimensions of frailty has the potential to provide a more nuanced understanding of an individual’s state of health across multiple deficits than a single frailty score does. A decomposed approach to understanding frailty has the potential to be more meaningful to clinicians and patients, but further research is required to replicate findings and understand associations with outcomes.

## Data Availability

Data are available on reasonable request.
