# Supplementary material for: Frailty or frailties: exploring frailty index subdimensions in the English Longitudinal Study of Ageing
Source: J Epidemiol Community Health. 2024 Jul 23;78(10):609–15. doi: 10.1136/jech-2023-221829 (PMC11420715; doi:10.1136/jech-2023-221829)

Supplementary Material

Table 1: List of Deficits

ELSA Variable Name	Description
hemobwa	Difficulty walking 100m
hemobsi	Difficulty sitting 2 hrs
hemobch	Difficulty getting up from chair
hemobcs	Difficulty climbing several flights of stairs without resting
hemobcl	Difficulty climbing one flight of stairs without resting
hemobst	Difficulty stooping, kneeling or crouching
hemobre	Difficulty extending arms above shoulders
hemobpu	Difficulty pulling or pushing large objects
hemobli	Difficulty lifting or carrying weights over 10 pounds
hemobpi	Difficulty picking up a 5p coin
headldr	Difficulty dressing
headlwa	Difficulty walking across a room
headlba	Difficulty bathing
headlea	Difficulty eating, such as cutting up food
headlbe	Difficulty getting in and out of bed
headlwc	Difficulty using the toilet
headlma	Difficulty using map
headlpr	Difficulty preparing a hot meal
headlsh	Difficulty shopping for groceries
headlph	Difficulty making phone calls
headlme	Difficulty taking medications
headlhg	Difficulty doing housework / gardening
headlmo	Difficulty managing money
hedimbp	High blood pressure
hediman	Angina
hedimmi	Heart attack
hedimhf	Congestive heart failure
hedimar	Abnormal heart rhythm
hedimdi	Diabetes
hedimst	Stroke
hediblu	Lung disease
hedibas	Asthma
hedibar	Arthritis
hedibos	Osteoporosis
hedibca	Cancer

hedibpd	Parkinson's
hedibps	Psychiatric condition
hedibad	Alzheimer's
hedibde	Dementia
psceda	Depressed
pscedb	Felt everything was an effort
pscedc	Restless sleep
pscedd	Lack of happiness
pscede	Loneliness
pscedf	Lack of life enjoyment
pscedg	Sadness
pscedh	Could not get going much of the time
hehelf*	Self-reported general health
heeye*	Eyesight impairment
hehear*	Hearing impairment
hefla	Fall
hefrac	Hip fracture
heji	Joint replacement
mmpain	Pain whilst walking
cfdatd	Whether correct day of month given
cfdatm	Whether correct month given
cfdaty	Whether correct year given
cfday	Whether correct day given
cfmem	Memory prompt needed
cflisenq	Whether reaches cutoff for cflisen
cfaniq	Whether reaches cutoff for cfani
cflisdq	Whether reaches cutoff for cflisd
cflisen	Number of words recalled immediately
cfani	Number of animal mentioned
cflisd	Number of words recalled after delay

Unless otherwise indicated, deficits were binary (0 = no deficit is reported; 1 = deficit is reported).

*Ordinal variables were dichotomised from the reported 5-point Likert scale (Excellent, Very good, Good, Fair, Poor), where Fair or Poor were treated as deficit present). In determining the appropriate cut-off point for dichotomisation, we considered the prevalence of each deficit at various thresholds. It was evident that using large cut-off points resulted in overly saturated deficits. Therefore we opted for a middle ground to ensure a balanced representation of deficits.

Table 2: Prevalence of Deficits

ELSA Variable Name	Deficit Description	Prevalence		
		Men	Women	Total
hedibar	Arthritis	39.0%	56.4%	48.8%
hedimbp	High blood pressure	46.4%	44.6%	45.4%
hemobst	Difficulty stooping, kneeling or crouching	38.1%	50.3%	45.0%
pscedc	Restless sleep	33.0%	45.8%	40.3%
hemobcs	Difficulty climbing several flights of stairs without resting	28.7%	45.6%	38.3%
hefla	Fall	26.0%	29.8%	28.1%
hemobch	Difficulty getting up from chair	23.3%	29.9%	27.1%
hehelf	Self-reported general health	25.9%	27.3%	26.7%
hemobli	Difficulty lifting or carrying weights over 10 pounds	15.8%	33.9%	26.1%
hehear	Hearing impairment	31.0%	21.3%	25.5%
hemobpu	Difficulty pulling or pushing large objects	13.0%	25.4%	20.0%
headlhg	Difficulty doing housework / gardening	16.0%	22.0%	19.4%
pscedh	Could not get going much of the time	15.4%	21.7%	18.9%
pscedb	Felt everything was an effort	14.5%	21.9%	18.7%
hemobcl	Difficulty climbing one flight of stairs without resting	13.4%	21.4%	17.9%
pscedg	Sadness	10.9%	21.5%	16.9%
hemobwa	Difficulty walking 100m	14.3%	17.9%	16.4%
cfdatd	Whether correct day of month given	17.4%	15.5%	16.3%
headldr	Difficulty dressing	15.5%	15.3%	15.4%
hedimdi	Diabetes	16.8%	12.3%	14.3%
hedibos	Osteoporosis	4.5%	18.5%	12.4%
hemobsi	Difficulty sitting 2 hrs	9.7%	13.9%	12.1%
hemobre	Difficulty extending arms above shoulders	8.7%	14.3%	11.9%
headlsh	Difficulty shopping for groceries	8.4%	14.1%	11.7%
hedibas	Asthma	10.1%	12.8%	11.6%
headlba	Difficulty bathing	9.6%	12.3%	11.2%
hedimar	Abnormal heart rhythm	12.1%	9.7%	10.7%
pscede	Loneliness	6.8%	13.4%	10.5%
psceda	Depressed	6.6%	13.4%	10.5%
hedibca	Cancer	10.6%	9.4%	10.0%
hedibps	Psychiatric condition	5.3%	9.9%	7.9%
hemobpi	Difficulty picking up a 5p coin	7.3%	8.0%	7.7%
mmpain	Pain whilst walking	5.7%	9.2%	7.7%
pscedf	Lack of life enjoyment	4.4%	9.9%	7.5%
pscedd	Lack of happiness	5.0%	9.3%	7.5%
headlbe	Difficulty getting in and out of bed	5.8%	8.6%	7.4%

headlpr	Difficulty preparing a hot meal	5.5%	8.2%	7.0%
hediblu	Lung disease	7.7%	6.0%	6.8%
headlma	Difficulty using map	4.7%	7.9%	6.5%
headlmo	Difficulty managing money	5.3%	5.8%	5.6%
headlwa	Difficulty walking across a room	4.5%	5.6%	5.1%
headlwc	Difficulty using the toilet	3.5%	6.4%	5.1%
hedimst	Stroke	5.8%	4.3%	4.9%
headlme	Difficulty taking medications	4.7%	4.4%	4.5%
headlph	Difficulty making phone calls	5.0%	3.8%	4.3%
heji	Joint replacement	3.6%	4.8%	4.3%
heeye	Eyesight impairment	3.4%	4.7%	4.2%
hediman	Angina	4.1%	3.2%	3.6%
hedimmi	Heart attack	4.5%	2.7%	3.5%
headlea	Difficulty eating, such as cutting up food	2.9%	3.8%	3.4%
cfdaty	Whether correct year given	3.3%	3.3%	3.3%
hedibde	Dementia	2.8%	3.1%	3.0%
cfdatm	Whether correct month given	2.9%	2.8%	2.9%
cfday	Whether correct day given	1.5%	2.6%	2.1%
hedimhf	Congestive heart failure	1.6%	1.2%	1.4%
hedibpd	Parkinson's	1.8%	0.8%	1.2%
hefrac	Hip fracture	1.1%	1.1%	1.1%
hedibad	Alzheimer's	1.1%	1.0%	1.0%

Table 3: Factor Loadings

Deficit	Factor 1	Factor 2	Factor 3	Factor 4
Difficulty walking 100m	0.64	0.17	-0.03	-0.03
Difficulty sitting 2 hrs	0.49	-0.03	0.01	-0.10
Difficulty getting up from chair	0.67	-0.06	0.00	-0.03
Difficulty climbing several flights of stairs without resting	0.73	-0.09	0.01	0.09
Difficulty climbing one flight of stairs without resting	0.67	0.11	-0.01	0.01
Difficulty stooping, kneeling or crouching	0.69	-0.11	-0.01	0.04
Difficulty extending arms above shoulders	0.40	0.15	0.04	-0.11
Difficulty pulling or pushing large objects	0.66	0.11	0.03	-0.01
Difficulty lifting or carrying weights over 10 pounds	0.69	0.05	0.03	0.05
Difficulty picking up a 5p coin	0.22	0.27	0.01	-0.14
Difficulty dressing	0.44	0.28	0.02	-0.16
Difficulty walking across a room	0.24	0.47	-0.01	-0.19
Difficulty bathing	0.32	0.47	0.04	-0.14
Difficulty eating, such as cutting up food	0.05	0.53	0.03	-0.21
Difficulty getting in and out of bed	0.30	0.38	0.05	-0.25
Difficulty using the toilet	0.16	0.49	0.04	-0.26
Difficulty using map	0.00	0.60	0.05	0.01
Difficulty preparing a hot meal	0.10	0.72	0.03	-0.04
Difficulty shopping for groceries	0.36	0.48	0.06	-0.04
Difficulty making phone calls	-0.06	0.69	-0.02	0.03
Difficulty taking medications	-0.05	0.77	0.00	0.15
Difficulty doing housework / gardening	0.57	0.23	0.04	-0.01
Difficulty managing money	-0.02	0.75	0.04	0.19
High blood pressure	0.24	-0.12	0.01	0.07
Angina	0.16	-0.01	0.00	0.05
Heart attack	0.14	0.05	-0.03	0.06
Congestive heart failure	0.16	0.00	0.01	0.08
Abnormal heart rhythm	0.19	-0.06	0.02	0.09
Diabetes	0.19	-0.05	0.00	0.08
Stroke	0.14	0.11	0.00	0.06
Lung disease	0.26	-0.10	0.00	0.06
Asthma	0.18	-0.09	0.03	0.04
Arthritis	0.48	-0.23	0.02	0.02
Osteoporosis	0.20	-0.01	0.08	0.00
Cancer	0.07	0.00	0.03	-0.01
Parkinson's	0.06	0.11	-0.02	-0.04
Psychiatric condition	0.14	-0.05	0.16	-0.01

Alzheimer's	-0.13	0.42	0.02	0.14
Dementia	0.00	0.48	-0.03	0.29
Depressed	-0.05	0.01	0.72	-0.01
Felt everything was an effort	0.31	-0.03	0.47	0.00
Restless sleep	0.17	-0.12	0.26	-0.02
Lack of happiness	-0.10	0.01	0.65	-0.01
Loneliness	0.03	-0.01	0.50	0.03
Lack of life enjoyment	-0.02	0.04	0.59	0.01
Sadness	-0.03	-0.03	0.60	0.00
Could not get going much of the time	0.29	-0.02	0.40	0.05
Self-reported general health	0.59	-0.10	0.15	0.08
Eyesight impairment	0.10	0.27	0.03	-0.04
Hearing impairment	0.17	0.05	0.04	0.05
Fall	0.26	-0.01	0.06	0.06
Hip fracture	0.10	0.06	-0.02	0.04
Joint replacement	0.16	-0.09	-0.01	0.04
Pain whilst walking	0.30	-0.09	0.05	-0.05
Whether correct day of month given	0.14	0.07	0.02	0.32
Whether correct month given	0.11	0.12	0.00	0.54
Whether correct year given	0.13	0.18	0.00	0.55
Whether correct day given	0.04	0.18	0.02	0.45

Table 4: Literature Summary

Paper	Sarkisian et al (2008) [1]	Bielderman et al (2013) [2]	King-Kallimanis et al (2014) [3]	Bohn (2022) [4]
Data Source	MacArthur Study Waves 1 and 2 (1988-1992)	Local health authority survey of older adults (2008)	SHARE, Wave 1 (2004-2006)	Victoria Longitudinal Study
Geography	USA	the Netherlands	12 EU countries	Canada
Participants	Aged 70-79 years	Aged ≥ 65 years	Aged ≥ 50 years	Aged ≥ 53 years
N	1010	1508	27,938	649
Frailty Measure	CHS frailty phenotype	GFI frailty phenotype	Frailty phenotype	Multi-morbidity items
Number of Items in Measure	5	15	7	30
Item Types	Continuous and ordinal	Dichotomised	Dichotomised	Ordinal (treated as continuous)
Number of Frailty Dimensions	2	3	1	7
Frailty Dimensions	(1) Physical activity, slowness, weakness; (2) Weight loss and exhaustion	(1) Daily Activities; (2) Psychosocial Functioning; (3) Health Problems	Frailty	(1) Mobility; (2) Instrumental Health; (3) Emotional Wellbeing; (4) Comorbidity; (5) Respiratory Symptoms; (6) Cardiac Symptoms; Physical Activity
Frailty pro-files	Not examined	Not examined	Not examined	Not clinically frail; (2) Mobility-type frailty; (3) Respiratory- type frailty
Method	PCA	EFA	CFA	EFA
Model Fit	Not reported	Not reported	RMSEA <0.045; CFI >0.95	RMSEA =.03; CFI =.90
Explained Variance	48%	50.6%	Not reported	Not reported

Association with Out- comes	Regression models for 4-year disability and 9- year mortality	Not examined	Not examined	Risk for accelerated cognitive decline and impairment
Drivers Explored	Not examined	Age, education significant. Sex, living situation, financial status not significant.	Not examined	Age not examined. Sex not significant.

Abbreviations: SHARE, Survey of Health, Ageing and Retirement in Europe; CHS, Cardiovascular Health Study; GFI, Groningen Frailty Indicator; PCA, Principal Component Analysis; EFA, Exploratory Factor Analysis; CFA, Confirmatory Factor Analysis

Table 5: Frailty Indicators Used in the Prior Literature on the Dimensionality of Frailty

**Fried's Frailty Phenotype [3]**	**Cardiovascular Health Study (CHS) Frailty Criteria [1]**	**Groningen Frailty Indicator [2]**	**British Women's Heart and Health Study (BWHHS) Frailty Indicators [5]**	**Victoria Longitudinal Study (VLS) Frailty Index [4]**	**ELSA Frailty Index**
Because of health problem expected to last >3 months, do you have difficulty...	Timed 10-foot walk	Shopping	Have you had a fall in past year?	Health has affected ability to travel	Difficulty walking 100m
Walking 100 m?	Grip strength	Walking outdoors	Compared with your activity level 3 years ago, are you doing more, same or less?	Health has affected ability to socialize	Difficulty sitting 2 hrs
Climbing a flight of stairs without resting?	Energy expenditure–weighted assessments of engagement in recreational, exercise, housework, and yardwork activities	Dressing and undressing	Do you have problems washing or dressing? (no problem, some problem, unable to wash and dress)	Health has affected ability to do hobbies	Difficulty getting up from chair
Highest of 4 (2 from each hand) of dynamometer measurement of grip strength in kilograms	Percentage of body weight lost (or gained) between Waves 1 and 2	Going to the toilet	Is your present state of health causing you problems with household chores?	Health has affected ability to do mental activities	Difficulty climbing several flights of stairs without resting
How often do you engage in activities that require a low or moderate level of	During the past week, how much have you been distressed by feeling low in energy	Physical fitness	Difficulty in carrying out activity on their own: going up and downstairs	Health has affected ability to get around town	Difficulty climbing one flight of stairs without resting

energy such as gardening, cleaning the car or going for a walk?	or slowed down?				
What has your appetite been like?		Vision problems	Difficulty in carrying out activity on their own: Walking about	Health has affected ability to do chores	Difficulty stooping, kneeling or crouching
Have you been eating more or less than usual?		Hearing problems	Difficulty in carrying out activity on their own: Going out of the house	Bradburn negative affect (restless, lonely, bored, depressed, upset due to criticism)	Difficulty extending arms above shoulders
In the past month, have you had too little energy to do the things you want?		Unintentional weight loss	Do you have trouble with your eyesight? (not simply needing specs)	CES-D “during the past week, my sleep was restless”	Difficulty pulling or pushing large objects
		Use of more than three medicines	Compared to five years ago, is your memory: improved, same, almost as good, worse, much worse? Dementia on medical exam.	CES-D “during the past week, I felt depressed”	Difficulty lifting or carrying weights over 10 pounds
		Memory complaints	Your health over all: are you anxious or depressed, not depressed – moderately, extremely.	CES-D “during the past week, I felt lonely”	Difficulty picking up a 5p coin
		Experience of emptiness	Do you ever have any pain or discomfort in your chest?	Anemia	Difficulty dressing

		Missing people around	Have you ever had a severe pain across the front of your chest lasting for half an hour or more?	Sex-related health problems (i.e., gynecological problems or prostate problems)	Difficulty walking across a room
		Feeling abandoned	Do you usually bring up phlegm (spit) from your chest first thing in the morning in the winter?	Gastrointestinal problems (colitis/diverticulitis, gall bladder trouble, and/or liver trouble)	Difficulty bathing
		Feeling sad/dejected	In the past four years, have you ever had a period of increased cough and phlegm lasting for 3 weeks or more?	Kidney or bladder trouble	Difficulty eating, such as cutting up food
		Feeling nervous/anxious	Do you get short of breath with other people of your own age on level ground?	Feeling short of breath	Difficulty getting in and out of bed
			Have you ever been told by a doctor that you have or have had asthma?	Bronchitis or emphysema	Difficulty using the toilet
			Have you ever been told by a doctor that you have or have had bronchitis or emphysema?	Asthma	Difficulty using map
			Have you ever been told	Pulse pressure	Difficulty preparing a

		by a doctor that you have or have had arthritis?		hot meal
		Have you ever been told by a doctor that you have or have had high blood pressure?	Heart trouble	Difficulty shopping for groceries
		Have you ever been told by a doctor that you have or have had thyroid disease?	Hardening of arteries (i.e., atherosclerosis)	Difficulty making phone calls
		Have you ever been told by a doctor that you have or have had a cataract?	High blood pressure	Difficulty taking medications
		Have you ever been told by a doctor that you have or have had glaucoma?	Stroke	Difficulty doing housework / gardening
		Have you ever been told by a doctor that you have or have had depression?	Finger dexterity	Difficulty managing money
		Have you ever been told by a doctor that you have or have had diabetes?	Timed turn	High blood pressure
		Have you ever been told by a doctor that you	Grip strength	Angina

		have or have had gastric or peptic ulcer?		
		Have you ever been told by a doctor that you have or have had heart attack (MI)?	Use of walker, cane, or wheelchair	Heart attack
		Have you ever been told by a doctor that you have or have had angina?	Stay at home but in chair most of the time	Congestive heart failure
		Have you ever been told by a doctor that you have or have had a stroke?	Health has affected ability to do physical recreational activities	Abnormal heart rhythm
		Have you ever been told by a doctor that you have or have had cancer?	Spinal condition and/or back trouble	Diabetes
		Cardiovascular disease (diagnosed angina, MI, stroke)	Arthritis (rheumatoid and/or osteo)	Stroke
		Body mass index: high or low		Lung disease
		Postural hypotension: According to consensus definition		Asthma
		Hypertensive (>140/90)		Arthritis
		Waist hip ratio (>/<0.85		Osteoporosis
		Sinus tachycardia (>100		Cancer

			bpm)		Parkinson's<br>Psychiatric condition<br>Alzheimer's<br>Dementia<br>Depressed<br>Felt everything was an effort<br>Restless sleep<br>Lack of happiness<br>Loneliness<br>Lack of life enjoyment<br>Sadness<br>Could not get going much of the time<br>Self-reported general health<br>Eyesight impairment<br>Hearing impairment<br>Fall<br>Hip fracture<br>Joint replacement<br>Pain whilst walking<br>Whether correct day of month given<br>Whether correct month given<br>Whether correct year

					given<br>Whether correct day given<br>Memory prompt needed<br>Whether reaches cutoff for cflisen<br>Whether reaches cutoff for cfani<br>Whether reaches cutoff for cflisd<br>Number of words recalled immediately<br>Number of animal mentioned<br>Number of words recalled after delay

References

[1] Sarkisian, C. A., Gruenewald, T. L., John Boscardin, W., & Seeman, T.E. (2008). Preliminary evidence for subdimensions of geriatric frailty: the MacArthur study of successful aging. Journal of the American Geriatrics Society, 56(12), 2292-2297.

[2] Bielderman, A., van der Schans, C. P., van Lieshout, M. R. J., de Greef, M. H., Boersma, F., Krijnen, W. P., & Steverink, N. (2013). Multidimensional structure of the Groningen Frailty Indicator in community-dwelling older people. BMC geriatrics, 13, 1-9.

[3] King-Kallimanis, B. L., Kenny, R. A., & Savva, G. M. (2014). Factor structure for the frailty syndrome was consistent across Europe. Journal of clinical epidemiology, 67(9), 1008-1015.

[4] Bohn, L., Zheng, Y., McFall, G. P., & Dixon, R. A. (2021). Portals to frailty? Data-driven analyses detect early frailty profiles. Alzheimer's Research & Therapy, 13, 1-12.

[5] Kamaruzzaman, S., Ploubidis, G. B., Fletcher, A., & Ebrahim, S. (2010). A reliable measure of frailty for a community dwelling older population. Health and quality of life outcomes, 8, 1-14.

Figure 1: Scree Plot

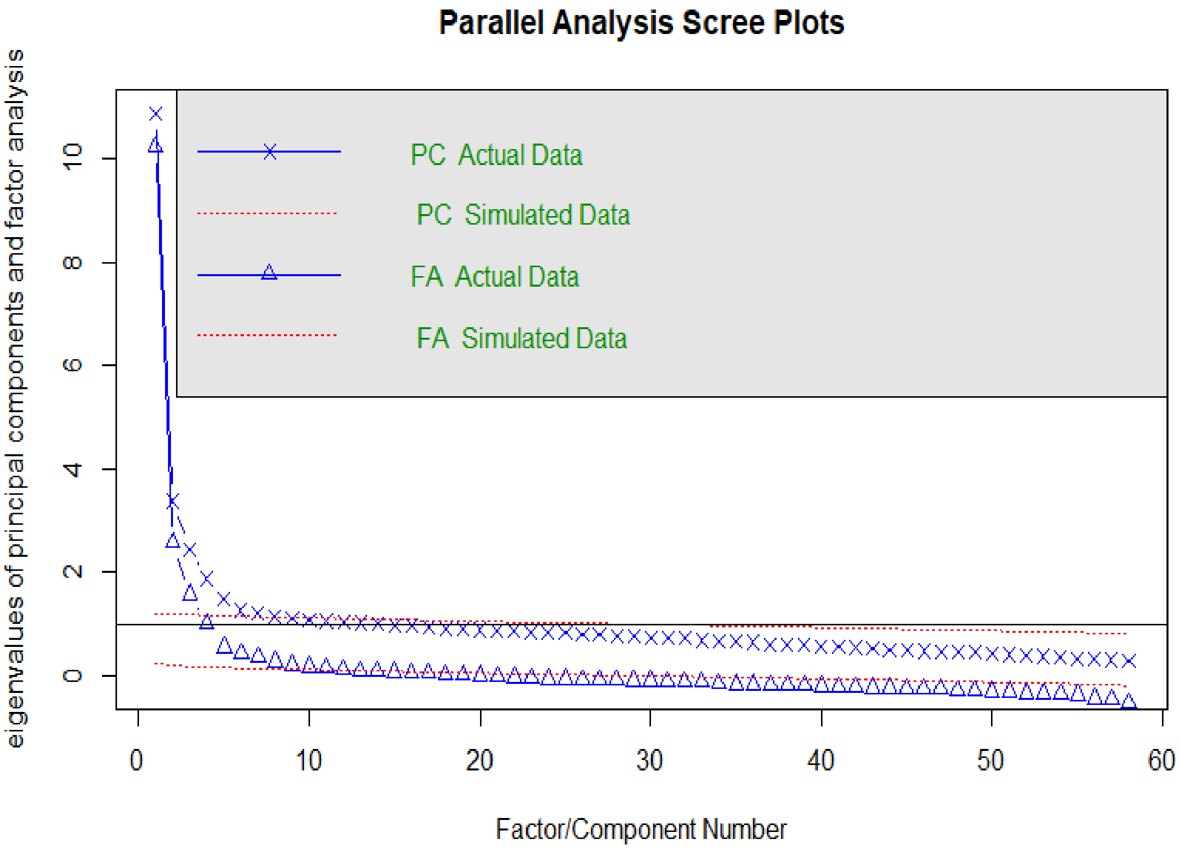

Figure 2. Correlations between Individuals’ Latent Factor Scores and Frailty Index Score

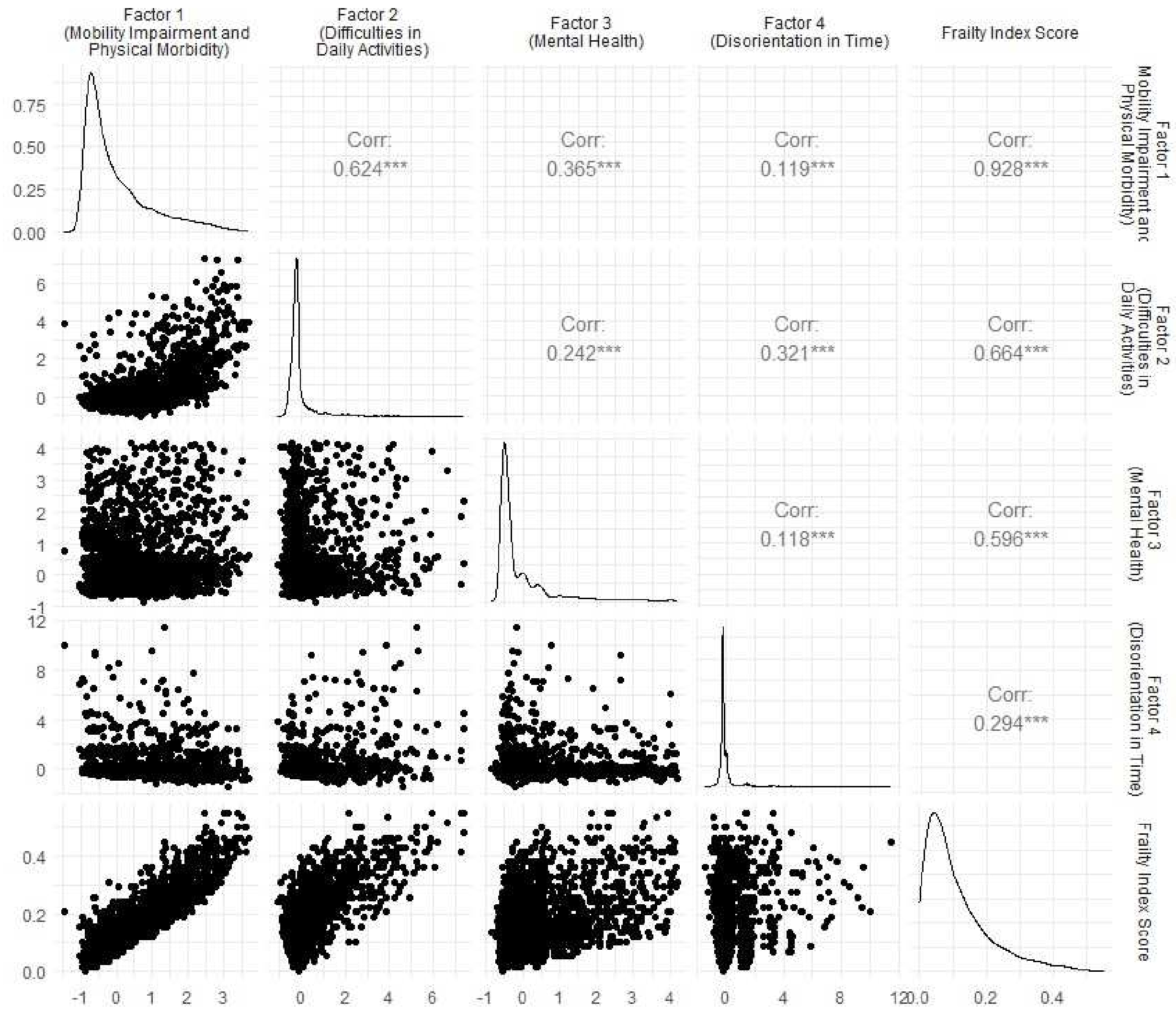

Supplement: Supplementary data [file jech-2023-221829supp001.pdf]
